# Coping with differences in snow cover: the impact on the condition, physiology and fitness of an arctic hibernator

**DOI:** 10.1093/conphys/cox065

**Published:** 2017-11-29

**Authors:** Michael J Sheriff, Rudy Boonstra, Rupert Palme, C Loren Buck, Brian M Barnes

**Affiliations:** 1 Department of Ecosystem Science and Management, The Huck Institute of Life Sciences, The Pennsylvania State University, University Park, PA 16802, USA; 2 Department of Biological Sciences, University of Toronto Scarborough, Toronto, ON M1C 1A4, Canada; 3 Department of Biomedical Sciences, University of Veterinary Medicine Vienna, Vienna 1210, Austria; 4 Center for Bioengineering Innovation, Northern Arizona University, Flagstaff, AZ 86001, USA; 5 Institute of Arctic Biology, University of Alaska Fairbanks, Fairbanks, AK 99775, USA

**Keywords:** Arctic ground squirrel, climate change, phenology, stress physiology

## Abstract

The Earth’s climate is changing at an unprecedented rate and, as ecologists, we are challenged with the difficult task of predicting how individuals and populations will respond to climate-induced changes to local and global ecosystems. Although we are beginning to understand some of the responses to changing seasonality, the physiological mechanisms that may drive these responses remain unknown. Using long-term data comparing two nearby populations (<20 km apart) of free-living arctic ground squirrels in northern Alaska, we have previously shown that the timing of spring snowmelt greatly influences their phenology of hibernation and reproduction in a population and site-specific manner. Here, we integrate these site-specific phenologies with body condition, stress physiology, reproductive success and juvenile recruitment to understand phenotypic selection in the two populations. We found that at the site with relatively late spring snowmelt and early autumn snow cover: (i) adult females were larger and in better body condition but had significantly higher stress hormone levels; (ii) females had similar numbers of comparably sized offspring, but offspring had higher stress hormone levels; and (iii) offspring density was lower just prior to hibernation. Thus, adult females at the two sites appear to use different coping strategies that allow them to maintain reproductive fitness; however, marked shortening of the active season because of later snowmelt in spring and earlier snow cover in autumn may compromise juvenile recruitment. We discuss the significance of these findings within the broader context of changing animal-environment relationships.

## Teaser

This study compares two nearby populations of arctic ground squirrels that experience significant differences in the timing of snowmelt and snow cover relative to their phenologies of hibernation and breeding. We found differences in the body condition, stress physiology and reproductive success of adult females, and in the subsequent juvenile recruitment between the populations.

## Introduction

How organisms interact with their biotic and abiotic environments critically influences their survival and reproductive success. Understanding these interactions, their mechanisms and their influence on fitness has taken on new urgency as climate change is rapidly altering environmental conditions and seasonality (ACIA, 2005; IPCC, 2014). Animal and plant phenologies (recurring seasonal events) are among the best-studied response traits to changing environments and seasonality, and it is now evident that climate change can result in mismatches, or loss of alignment, between the timing of animal breeding and food presence and abundance. This mismatch may translate into negative effects on fitness and population dynamics (Visser *et al.*, 1998; Parmesan and Yohe, 2003; Both *et al.*, 2006, 2010; Saino *et al.*, 2011). For example, in Western Greenland, the timing of caribou (*Rangifer tarandus*) calving has not kept pace with the advancement of the plant growing season and may have contributed to the observed decline in caribou reproductive success (Post and Forschhammer, 2008). In the Rocky Mountains, yellow-bellied marmots (*Marmota flaviventris*) have advanced their timing of emergence from hibernation and weaning of young in response to warming temperatures, although snowmelt date and thus the beginning of the plant growing season has not changed (Inouye *et al.*, 2000; Ozgul *et al.*, 2010). This has resulted in greater body mass of marmots prior to hibernation, decreased adult mortality and an increase in population size presumably because marmots are active for longer (Ozgul *et al.*, 2010). Although we are beginning to understand some of the behavioral responses to changing seasonality, the physiological mechanisms which drive these responses remain largely unknown (Pörtner and Farrell, 2008; Meylan *et al.*, 2012; Wingfield, 2013; Sheriff *et al.*, 2015).

One physiological integrative system that will play a key role in affecting the ability of animals to cope with changing environments is the hypothalamic-pituitary-adrenal (HPA) axis and subsequent secretion of glucocorticoids (Boonstra, 2004; Wikelski and Cooke, 2006; Wingfield, 2008, 2013; Meylan *et al.*, 2012; Boonstra *et al.*, 2014; Dantzer *et al.*, 2014). The HPA axis and release of glucocorticoids play a critical role in how animals integrate, cope with, and respond to changes and challenges in their environment (Wingfield *et al.*, 1998; Sapolsky *et al.*, 2000). These hormones are closely tied to individual performance and fitness, and can be influenced by a number of environmental factors including weather, food availability and predation risk (Boonstra *et al.*, 1998; Romero *et al.*, 2000; Buck *et al.*, 2007; Breuner *et al.*, 2008; Bonier *et al.*, 2009; Kitaysky *et al.*, 2010; Clinchy *et al.*, 2013). They are important for shaping the behavior and morphology of individuals, can alter population and community dynamics through their impact on survival and reproduction, and can have generational consequences shaping the development and phenotype of offspring (Boonstra, 2004, 2013; Meylan *et al.*, 2012; Satherthwaite *et al.*, 2012; Sheriff *et al.*, 2017). Ultimately, investigating glucocorticoid levels may provide a key window into individual health and fitness, and population dynamic consequences for animals facing a changing environment.

Our objective was to compare the body condition, stress physiology, reproductive success and recruitment of arctic ground squirrels (AGS; *Urocitellus parryii*) living in two populations that experience significant differences in the relationship between snow cover and adult female phenology. Using long-term data comparing two nearby populations of free-living AGS in northern Alaska, we have previously shown that at our Atigun site, where snowmelt occurs on average 26 days earlier than at our Toolik site, female AGS emerge from hibernation and give birth significantly earlier than females living at Toolik, <20 km away (Sheriff *et al.*, 2011a). However, relative to the timing of when snowmelt occurs, Atigun females have shifted their phenology of annual events significantly later; they emerge from hibernation 2 weeks before snowmelt and give birth 2 weeks after complete melt, whereas Toolik females emerge from hibernation 4 weeks before snowmelt and give birth coincident with complete melt (Sheriff *et al.*, 2015). Thus, the timing of key AGS life history events is not synchronous with the environmental differences between the sites. Although AGS are generalist herbivores and will eat roots, seeds, old berries and dried leaves in spring, ground squirrels will not dig beneath snow to access food (Bronson, 1980; Murie and Harris, 1982; Sheriff *et al.*, 2011a; Williams *et al.*, 2014). Thus, Toolik females face harsher spring conditions than Atigun females; they must deal with prolonged spring snow cover and the associated reduction in food availability, having to sustain their entire pregnancy during this time. Given this significant difference in their phenological-environmental relationship, we predicted that, compared with Atigun females: (i) Toolik females would be in worse body condition and have higher glucocorticoid levels; (ii) Toolik females would have lower fitness, as measured by number of offspring per female and by offspring condition; and (iii) recruitment (juvenile density at hibernation) would be lower at Toolik.

## Materials and methods

### Study animal

Arctic ground squirrels are distributed across northern Alaska, Canada, and eastern Siberia and are the northern most hibernator (Naughton, 2012). They live in generally well-drained sites in the tundra, the alpine and the meadows of the boreal forest. They are key prey for many predators, including golden eagles, hawks, owls, foxes, wolves, weasels, bears, wolverines and lynx. Their annual cycle includes a short 3–6 months of above-ground active season with spring breeding occurring immediately after females emerge from hibernation in mid to late April (Buck and Barnes, 1999a; Sheriff *et al.*, 2011a). The breeding season lasts only 2 weeks, and females exhibit behavioral estrus on only 1 day (Lacey *et al.*, 1997). Females produce a single litter ~25 days later in mid to late May, in underground burrows, and young appear above ground shortly before weaning in late June to early July (Lacey, 1991). Dispersal occurs 2–3 weeks thereafter, with nearly all males dispersing (~515 m) from their natal home range and most females remaining within their natal home range (~120 m from natal burrow; Byrom and Krebs, 1999). Adult females enter hibernation as early as late July, juvenile females enter hibernation in mid to late September, and juvenile males enter in early October (Sheriff *et al.*, 2011a). Animals spend the remaining 6–9 months sequestered in their hibernacula alternating between long bouts (2–3 weeks) of torpor at core body temperatures as low as −2.9°C and short (<1 day) periods of high body temperature (~36°C; Barnes, 1989; Buck *et al.*, 2008). During this long hibernation, females lose approximately 1 g of body mass per day and emerge from hibernation at their lowest body condition, having lost approximately 35% of their body mass including a 66% loss in body fat and a 21% loss in lean mass (Buck and Barnes, 1999b). Thus, at the most energetically expensive time (breeding) females are at their worst body condition.

### Study area and AGS phenological-environmental relationship

Our study occurred in 2011 and 2012 at two sites with large contiguous AGS populations, Toolik Lake and Atigun River. These sites are separated by approximately 20 km along the Dalton Highway in northern Alaska (68°N, 149°W). The topography is similar at both sites, being relatively flat with gently rolling hills underlain by continuous permafrost with a seasonal thaw depth of 1–2 m (Buck and Barnes, 1999b). Entrances to burrows at the two sites do not differ in exposure to or shelter from solar radiation, shade or wind.

Arctic ground squirrels are generalist herbivores (Batzli and Sobaski, 1980; Hobbie *et al.*, 2017) and, although there may be vegetation differences between the sites in terms of plant phenology and composition (albeit slight MJS pers. obs.; Toolik Environmental Data Center http://toolik.alaska.edu/edc/), the most pronounced difference between the sites is the marked food restriction in spring due to prolonged snow cover throughout AGS pregnancy at Toolik as compared to Atigun (Sheriff *et al.*, 2015). As snow melts at both sites it creates a mottled patchy environment, where most areas have snow-free and snow-covered patches. This patchy environment may present several challenges as AGS must traverse across snow patches to access snow-free areas, making squirrels more vulnerable to predation and increasing their cost of locomotion. Additionally, at Toolik 100% snow cover occurs from mid to late September, a critical time for offspring that are still fattening in preparation for hibernation (which begins late September—early October). At Atigun, 100% snow cover usually begins late September—early October after offspring have entered hibernation (Sheriff *et al.*, 2015).

The timing of snow cover has been recorded since 2007 using a camera (Campbell Scientific, CC640 Digital Camera) mounted on a tower facing across each study area that captures a daily image at solar noon. We assessed the percentage of area within each image covered with snow from April 15th (prior to first female emergence at either site) until October 10th (after offspring entrance into hibernation) for 2011 and 2012 from camera images.

### Animal handling and population estimates

Arctic ground squirrels were captured using live-traps (Tomahawk Live Trap Co., Tomahawk, Wisconsin) baited with carrot. Adult females were captured 10–14 days after conception at mid-gestation (early to mid-May) which we term gestation, and at weaning (early to mid-July); juveniles were captured at weaning, and prior to entering hibernation (early to mid-September) which we term pre-hibernation. Adult females were not trapped prior to entering hibernation as this can occur as early as late July and continue into late August, making standardization of timing difficult at a population level. Phenological differences between the sites were accounted for and, thus, AGS at Toolik were captured 6–10 days after those at Atigun. At each time point, we estimated population density via mark-recapture methods on 6 ha grids with stations spaced 20 m apart in a 15 × 10 m array and traps at alternate stations. Traps were set at 0800–1000 h depending upon season, and checked every 2 h, with no more than 4 capture sessions per day per site, and a total of eight capture sessions per site over a 3-day period. Upon first capture, AGS were weighed with a Pesola spring scale (±5 g), assessed for sex and ID, measured for zygomatic arch width (± 0.5 mm; as an index of body size), and assessed for reproductive condition. Upon initial capture, new individuals were uniquely ear-tagged (Monel #1 and a unique 4-color combination). A fecal sample, for later determination of cortisol metabolite levels, was collected only from individuals captured in the first session of the day and who had not been captured within the previous 48 h. These grids were placed within the larger contiguous populations at both sites.

‘Body condition’ was estimated from 86 adult females (31 from Toolik and 55 from Atigun) and 112 juveniles (41 from Toolik and 71 from Atigun) as the residuals of body mass (g) regressed on zygomatic arch width (a reliable estimate of skeletal size; Schulte-Hostedde *et al.*, 2005) with simple linear regression using ordinary least squares. Schulte-Hostedde *et al.* (2005) assessed techniques for determining body condition and report that this method performs best. ‘Reproductive output’ was estimated as the number of offspring per adult female (calculated from the density of each when young first appeared above ground). ‘Recruitment’ was estimated as the density of young-of-the-year (juveniles) at pre-hibernation. All density estimates were calculated using the maximum likelihood spatial model within the program Density version 5.02 using all the default parameters (Efford *et al.*, 2009). This model has been used to estimate AGS population density previously (Werner *et al.*, 2015).

### Stress physiology

We used an enzyme immunoassay to measure fecal cortisol metabolite (FCM) concentrations (reflecting glucocorticoid levels) from 41 adult females (18 from Toolik and 23 from Atigun) and 61 juveniles (24 from Toolik and 37 from Atigun), validated specifically for use with AGS (see Sheriff *et al.*, 2012 for details). FCM values represent one of the least invasive measures of physiological stress and have been widely used in wild animals (Sheriff *et al.*, 2011b; Dantzer *et al.*, 2014). FCM values reflect free (unbound to corticosteroid binding globulin) glucocorticoid levels found in the blood (Sheriff *et al.*, 2010; Fauteux *et al.* 2017) that an individual has experienced over a specific time period (Palme *et al.*, 2005), 4–12 h in AGS (Sheriff *et al.*, 2012). Thus, FCM values provide an integrated measure of circulation glucocorticoid levels, better reflecting the average physiological state of the individual as compared with blood samples, that provide a point-in-time sample (Sheriff *et al.*, 2011b). FCM values have been used to investigate the influence of both predictable (changes in season, reproductive status, etc.) and unpredictable stressors (predation risk, food limitations, competition) in a wide variety of free-living animals (reviewed in Sheriff *et al.*, 2011b; Dantzer *et al.*, 2014). Although it is often assumed that individuals with higher FCM levels have reduced fitness, it is important to appreciate that, as with any measure of physiological stress, the FCM—fitness relationship likely forms a bell shaped curve with greater levels potential increasing an individual’s ability to cope with a particular stressor to a certain threshold at which point greater levels reduce fitness (Selye, 1936). Thus, FCM levels are best suited to provide insights into how individuals cope with a particular stressor and should be paired with actual fitness measures (*sensu*Dantzer *et al.*, 2014).

In this study fecal samples were obtained within 2 h of capture, thus the stress of capture would not have affected measured FCM levels (Sheriff *et al.*, 2012). Further, although human disturbance may increase FCM values, trapping and sampling procedures were similar between sites and this potential disturbance likely did not affect potential site differences in FCM levels. Further, AGS are highly re-trappable (even within the same session), indicating humans and traps are not perceived as much of a threat. Upon collection in the field, feces were immediately placed on wet ice, transported back to the Toolik Field Station, and frozen at −20°C within 7 h of collection. Samples were transported on dry ice to the University of Toronto where they were lyophilized (LabConco, Missouri, USA) and homogenized. Following extraction with 80% methanol (50 ± 3 mg feces with 1 ml), FCMs were measured as outlined by Sheriff *et al.* (2012) using the 11-oxoetiocholanolone-EIA developed by Möstl *et al.* (2002). Intra-assay and inter-assay coefficient of variation (CV) were 15.3% and 14.6%, respectively (*n* = 22 plates).

### Statistical analyses

All data are presented as mean ± SE, unless otherwise stated. All statistics were performed using the software package STATISTICA 10. Because individual ground squirrels could not be tracked throughout the entire study, individual ID was tested as a covariate. The latter was found to be non-significant and was dropped from the models. The assumption of normality was tested with Shapiro–Wilks test, and the assumption of homogeneity of variances was tested with Levene’s test. For data that met the assumptions we used a 3-way ANOVA (site × date × year). For data that did not meet the assumptions a Kruskal–Wallis non-parametric test was used; no interaction effects could be tested. Comparisons of the means were considered significant if *P* < 0.05.

## Results

### Phenology and snow cover

**Figure 1:**
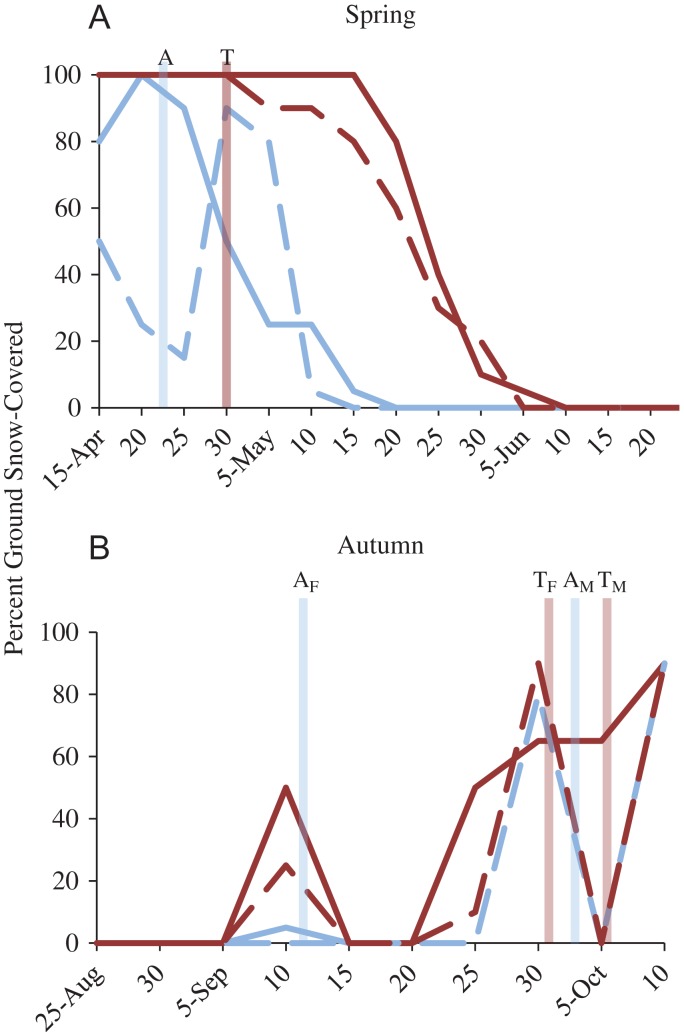
Percent of ground covered with snow at our Atigun (light blue) and Toolik (dark red) sites in 2011 (solid lines) and 2012 (hashed lines) in northern Alaska. (**A**) In spring, Atigun females (light blue, A) emerged from hibernation on April 22 ±2 d and Toolik females (red, T) on April 30 ± 2 d (average of both years). (**B**) In autumn, Atigun female offspring (light blue, A_F_) entered hibernation on September 12 ±2 d and male offspring (light blue, A_M_) on October 1 ± 5 d and at Toolik female offspring (red, T_F_) entered hibernation on September 18 ±2 d and male offspring (red, T_M_) on October 5 ± 4 d, respectively.

In the two springs combined, Atigun females emerged on April 22nd ± 2 days, 4 days before 50% snowmelt and 18–23 days before complete snowmelt, whereas Toolik females emerged on April 30th ± 2 days, 20–25 days before 50% snowmelt and 30–35 days before complete snowmelt (Fig. 1). At Toolik first onset of snow cover occurred between September 5th and 15th and covered between 25% and 50% of the ground, snow cover occurred again September 20–25th and by September 30th 65–90% of the ground was covered. At Atigun, first onset of snow cover occurred September 28th and by September 30th 90% of the ground was covered (data in 2011 were not collected past September 15th due to camera malfunction, however, no snow cover had occurred by this point).

### Adult female condition

#### Body size

**Figure 2:**
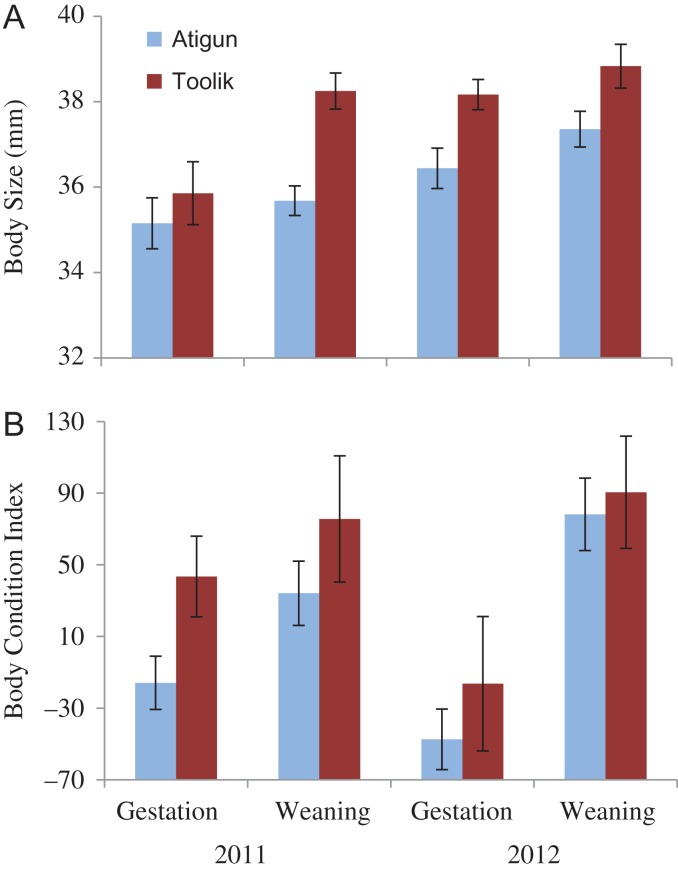
The body metrics (mean ± SE) of adult female arctic ground squirrels at Atigun (light blue) and Toolik (dark red) sites in northern Alaska: (**A**) body size (zygomatic arch width) and (**B**) body condition index (the residuals of mass regressed over body size) at mid-gestation (10–14 d after conception) and weaning.

We found an effect of site (F_1,78_ = 16.05, *P* < 0.0001), date (F_1,78_ = 9.55, *P* = 0.003) and year (F_1,78_ = 13.51, *P* < 0.0001), but no interaction effects (*P* > 0.05). Atigun females were smaller than Toolik females (zygomatic width = 36.2 ± 0.3 mm vs. 37.9 ± 0.3 mm, respectively; Fig. 2A). At both sites, females were smaller at the ~mid-point of gestation than at weaning (zygomatic width = 36.3 ± 0.3 vs. 37.4 ± 0.3 mm, respectively) and were smaller in 2011 than in 2012 (zygomatic width = 36.0 ± 0.3 vs. 37.5 ± 0.3 mm, respectively; Fig. 2A).

#### Body condition index

We found an effect of site (F_1,78_ = 4.32, *P* = 0.04) and of date (F_1,78_ = 20.54, *P* < 0.0001), but not of year (F_1,78_ = 0.22, *P* = 0.64) and an interaction effect between year*date (F_1,78_ = 4.69, *P* < 0.03) but no other interaction effect (*P* > 0.05). Average body condition of Atigun females was 2.5 times lower at gestation and 1.5 times lower at weaning than of Toolik females (Fig. 2B). At both sites, body condition increased from gestation to weaning (Atigun: −33.7 to 58.8, Toolik: 9.8 to 84.5, respectively; Fig. 2B). The interaction effect of year*date occurred because females had better gestation body condition but poorer weaning body condition in 2011 than in 2012 (Fig. 2B).

#### Fecal cortisol metabolites

**Figure 3:**
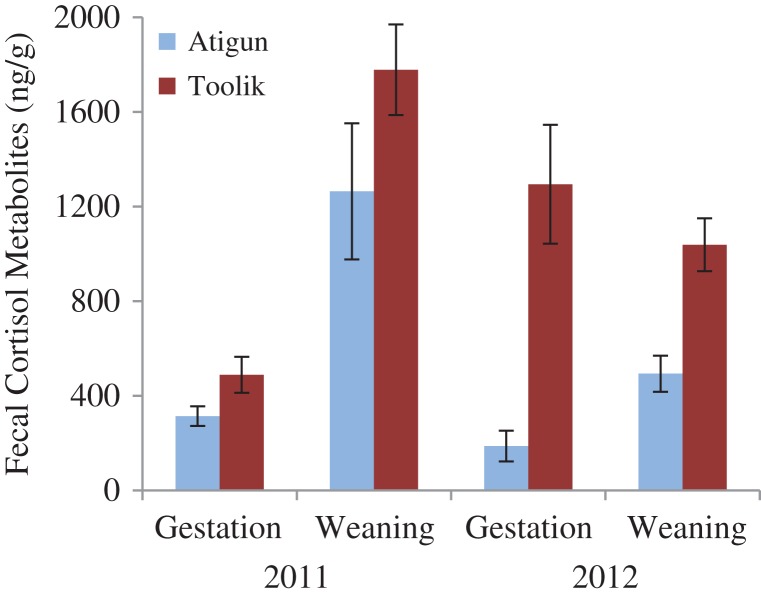
The glucocorticoid levels (fecal cortisol metabolites ng/g, mean ± SE) of adult female arctic ground squirrels at mid-gestation (10–14 d after conception) and at weaning at Atigun (light blue) and Toolik (dark red) sites in northern Alaska.

We found an effect of site (*Z* = −3.43, df = 23, 18, *P* = 0.0006) and date (*Z* = 2.91, df = 21, 20, *P* = 0.004), but not year (*Z* = −1.25, df = 23, 18, *P* = 0.21). Average FCM levels of Atigun females were 4.5 times lower at gestation and 1.5 times lower at weaning than Toolik females (Fig. 3). At both sites, FCM levels increased from gestation to weaning (Atigun: 233 ± 47 ng/g to 942 ± 201 ng/g, Toolik: 1025 ± 212 ng/g to 1366 ± 163 ng/g; Fig. 3).

#### Reproductive output

**Figure 4:**
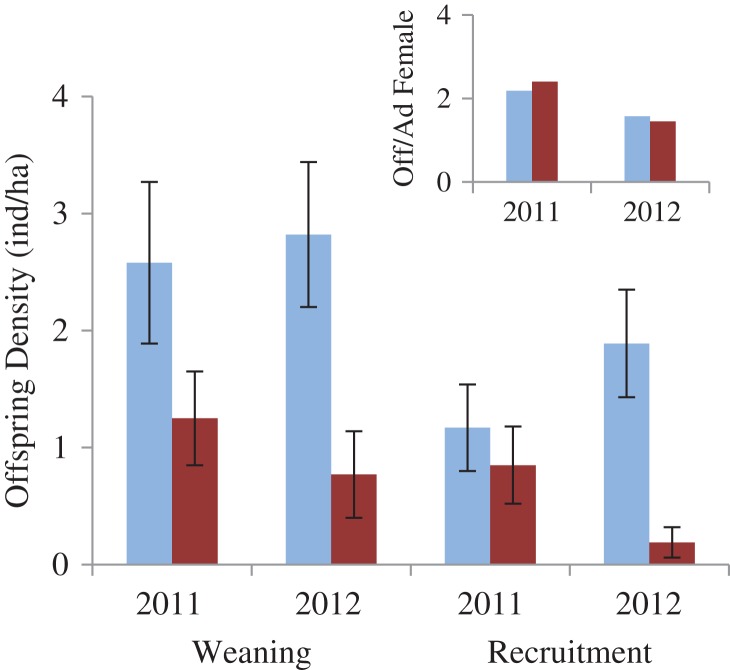
Arctic ground squirrels offspring density (mean ± SE) at weaning and at recruitment (pre-hibernation in early September) at Atigun (light blue) and Toolik (dark red) sites in northern Alaska. Inset shows number of offspring per adult female at weaning at Atigun (light blue) and Toolik (dark red).

The number of offspring/female at weaning was similar between the sites (Fig. 4 inset). Atigun females averaged 0.2 offspring/female fewer in 2011, and 0.1 offspring/female more in 2012 than Toolik females. Because of a higher adult female density (>1 ind/ha), juvenile density was higher at Atigun than at Toolik in both years of the study (2011: > 1.25 offspring/ha more, 2012: > 2 offspring/ha more at Atigun than Toolik; Fig. 4).

### Offspring condition

#### Body size

**Figure 5:**
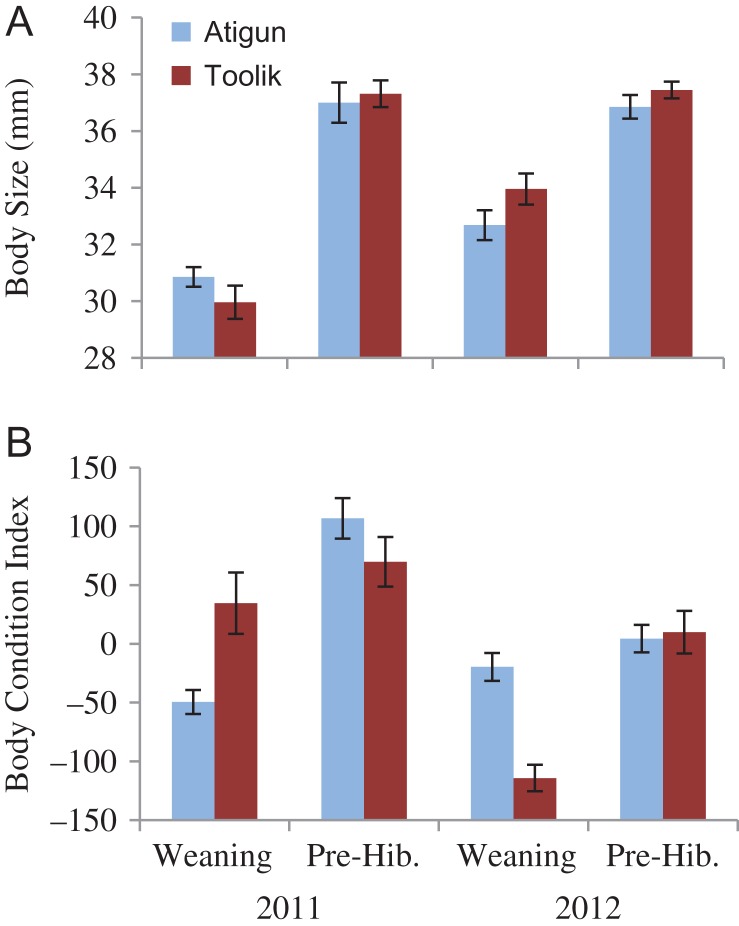
The body metrics (mean ± SE) of arctic ground squirrel offspring at Atigun (light blue) and Toolik (dark red) sites in northern Alaska: (**A**) body size (zygomatic arch width) and (**B**) body condition index (the residuals of weight regressed over body size) at weaning and at pre-hibernation.

We found an effect of year (*Z* = 8.39, df = 52, 60, *P* < 0.0001) and date (*Z* = 2.66, df = 66, 46, *P* = 0.008), but not site (*Z* = -0.02, df = 71, 41, *P* = 0.98). Thus, we found no difference in offspring body size between the two sites at either weaning or pre-hibernation (Fig. 5A). Offspring were smaller at weaning than just before they entered hibernation (zygomatic width = 31.9 ± 0.3 mm vs. 37.1 ± 0.3 mm, respectively; Fig. 5A). Offspring were smaller in 2011 than in 2012 (zygomatic width = 33.2 ± 0.6 mm vs. 35.1 ± 0.4 mm, respectively; Fig. 5A).

#### Body condition index

We found an effect of year (*Z* = 4.78, df = 52, 60, *P* < 0.0001) and date (*Z* = −3.07, df = 66, 46, *P* = 0.002), but not site (*Z* = 0.26, df = 71, 41, *P* = 0.80). Thus, we found no difference in offspring body condition between the two sites at either weaning or pre-hibernation (Fig. 5B). Offspring body condition increased from weaning to pre-hibernation (−32.2 ± 9.9 vs. 37.1 ± 9.8, respectively; Fig. 5B). Offspring were in better body condition in 2011 than in 2012 (32.4 ± 12.9 vs. −22.6 ± 8.3, respectively; Fig. 5B).

#### Fecal cortisol metabolites

**Figure 6:**
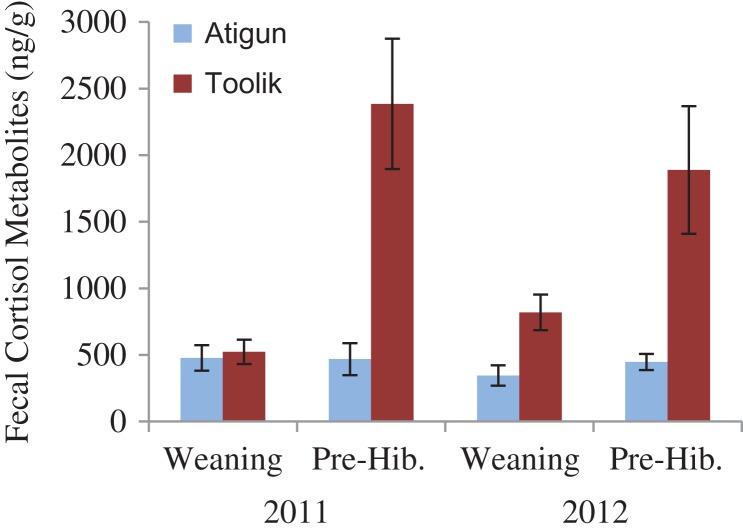
The glucocorticoid levels (fecal cortisol metabolites, ng/g, mean ± SE) of arctic ground squirrel offspring at weaning in early July and at pre-hibernation in September at Atigun (light blue) and Toolik (dark red) in northern Alaska.

We found an effect of site (F_1,53_ = 39.24, *P* < 0.0001) and date (F_1,53_ = 23.85, *P* < 0.0001), but not year (F_1,53_ = 0.32, *P* = 0.57), an interaction effect of site*date (F_1,53_ = 21.04, *P* < 0.0001), but no other interaction effects (*P* > 0.05). Atigun offspring averaged 3 times lower FCM levels than Toolik offspring: at weaning levels were 1.5 times lower and at pre-hibernation levels were 4.5 times lower in Atigun offspring than in Toolik offspring (Fig. 6). FCM levels increased from weaning until just prior to hibernation; however, these differences were driven by offspring at Toolik not by those at Atigun (offspring FCM levels from weaning to pre-hibernation; Atigun (*n* = 19, 18): 415 ± 62 ng/g to 454 ± 54 ng/g, Toolik (*n* = 12, 12): 720 ± 100 ng/g to 2137 ± 335 ng/g, respectively; Fig. 6).

#### Recruitment

We found that the density of juveniles (individuals/ha) at pre-hibernation was greater at Atigun than at Toolik, particularly in 2012 (2011: 1.17 ± 0.37 vs. 0.85 ± 0.33; 2012: 1.89 ± 0.46 vs. 0.19 ± 0.13, respectively; Fig. 4). We also found from weaning to hibernation juvenile density decreased 54% at Toolik but only 44% at Atigun.

## Discussion

We investigated how marked differences in the relationship between seasonality (timing of snowmelt in spring and snow cover in autumn) and phenology affected the body condition, stress physiology and reproductive success of adult females, and the subsequent recruitment of juveniles just prior to hibernation in two nearby AGS populations in northern Alaska. Because female squirrels living at Toolik experienced prolonged spring snow cover and hence lower food availability during pregnancy relative to those at Atigun (Fig. 1), we predicted that: (i) Toolik females would be in poorer body condition and have higher FCM levels (indicative of glucocorticoid levels); (ii) that they would have lower reproductive fitness; and (iii) that juvenile recruitment would be lower. However, we found that: (i) adult females at Toolik were larger and in better body condition but had significantly higher FCM levels during pregnancy and at weaning compared to Atigun females (Figs 2 and 3); (iia) the number of offspring produced per adult female and offspring body size and condition was similar between the two the sites (Figs 4 and 5); (iib) offspring FCM levels were higher at Toolik than at Atigun at weaning (by 1.5 times) and pre-hibernation (by 4.5 times), and levels nearly tripled in Toolik offspring from weaning to pre-hibernation whereas in Atigun offspring levels remained constant (Fig. 6); and (iii) juvenile density at hibernation was lower at Toolik than Atigun, and from weaning to hibernation density decreased 54% at Toolik but only 44% at Atigun (Fig. 4), indicating that recruitment is lower at Toolik. While this study demonstrates correlative results among phenotypic and life history traits between two populations of AGS that have a significantly different phenological-environmental relationship, we believe our findings may point to possible mechanistic relationships of how organisms can cope with and respond to environmental change. Below we discuss why our predictions did not match our results and discuss the significance of our findings within the broader context of climate change.

### Adult female condition

At Toolik, females emerged from hibernation to a snow-covered environment approximately 1 month prior to complete spring snowmelt, whereas at Atigun, females emerged to a partially snow-covered environment, 2 weeks prior to complete melt. Thus, the entire gestation period (~26 days) of Toolik females occurred prior to complete snowmelt, whereas Atigun females gave birth 2 weeks after complete melt. Given that access to food is limited for AGS by snow cover and females do not cache food, we expected Toolik females to be in worse condition in spring. Previously, we have shown that Toolik females lose mass for the first 1–2 weeks following emergence (we do not have data on Atigun females; Buck and Barnes, 1999a). We found, however, that Toolik females were larger and in better body condition than those at Atigun during pregnancy (10–14 days after emergence at mid-gestation) and at weaning. Ozgul *et al.* (2010) also found in another hibernator, the yellow-bellied marmot, that earlier emergence relative to snowmelt date resulted in larger individuals. The authors suggest this is due to a greater amount of time animals had to grow during their active season. In our system adult females are active on the surface for a similar amount of time between the sites, 119 ± 2 d/yr at Toolik and 123 ± 3 d/yr at Atigun (Sheriff *et al.*, 2011a) and thus we cannot attribute the larger size and better body condition of Toolik females to a longer growing or active season. If anything Toolik females have a more limited ability to invest in growth given that they emerge and go through gestation with relatively reduced food availability. Previously, we have found that, independent of sex, over time AGS increase mass, such that a 3-year-old weighs more than a 2-year-old; although we do not have morphometric data on size (Buck and Barnes, 1999a). Thus, one possible explanation is that females we sampled at Toolik are older than those we sampled at Atigun.

Toolik females also had significantly higher glucocorticoid levels (as indicated by FCM levels) during pregnancy and at weaning compared to those at Atigun. There are two non-exclusive explanations for these elevated levels. First, glucocorticoids play a principle role in mobilizing energy reserves, such as glucose (Boonstra *et al.*, 1998; Sapolsky *et al.*, 2000). Hence, it is possible that Toolik females had elevated glucocorticoid levels to facilitate gluconeogenesis, tissue catabolism and mobilization of internal reserves to meet the demands of the growing offspring in a poor environment, whereas Atigun females can meet their demands by foraging. Increased glucocorticoid levels may also stimulate foraging and increase feeding efficiency (Montanholi *et al.*, 2013), and thus aid Toolik females in food intake during foraging. Second, a number of external factors can also influence individual glucocorticoid levels including inclement weather (Romero *et al.*, 2000), reduced food availability (Kitaysky *et al.*, 2010) and increased predation risk (Clinchy *et al.*, 2013). For example, Sheriff *et al.* (2012) found no clear, single explanation for different glucocorticoid hormone patterns among AGS populations living in the southwestern Yukon. They hypothesized that glucocorticoid levels were influenced by a combination of differences in seasonal climate, adverse weather, density and visibility (a proxy for predation risk). It is likely that Toolik females also face a combination of factors in spring. The prolonged snow cover and reduced food availability throughout pregnancy could: (i) cause females to have elevated glucocorticoid levels to increase gluconeogenesis and internal energy mobilization, (ii) increase their glucocorticoid levels in response to a food restricted spring environment and (iii) force them to engage in riskier foraging behavior, as they roam further from the safety of their burrows in search of foraging areas. Although we do not have quantifiable data, red foxes and birds of prey are very often seen at both sites and there are active fox dens nearby both sites, thus we predict that the number of predators is very similar between the sites and, as such, greater foraging activity likely increases the potential for predation. An individual’s physiological state may have a great impact on their ability to cope with and respond to changes in the environment (Boonstra *et al.*, 1998; Wingfield *et al.*, 1998). It may also provide a good indication of an individual’s overall health (Romero, 2004; Dantzer *et al.*, 2014). Generally, those individuals with higher glucocorticoid levels are considered to be experiencing poor and unpredictable environmental conditions, and to be in worse condition and have reduced reproduction and survival (Wingfield *et al.*, 1998; Romero, 2004; Buck *et al.*, 2007; Dantzer *et al.*, 2014; but see Bonier *et al.*, 2009).

Our results appear paradoxical given that Toolik females are in better condition based on their weight and size, but in worse condition based on their FCM levels. Although we cannot rule out age as a possible factor influencing female weight and size, we suggest that the environments AGS experience may be driving factors in the differences we found. The later snowmelt and poorer environment Toolik females face in spring may have selected for larger more robust phenotypes, i.e. smaller females in poor body condition may simply not survive in such an environment. Earlier snowmelt at Atigun may allow small, poor condition females to survive and reproduce because of the better spring environment and greater access to food. This is supported by the fact that our smallest females at Toolik had zygomatic arch sizes of 34 (2 females) and 35 mm (2 females), whereas the smallest females at Atigun had arch sizes of 30 (1 female), 31 (1 female), 32 (2 female), 33 (4 females), 34 (2 females), 35 (6 females). However, emerging to a poor spring environment coupled with the energetic and food requirements of breeding, females support litters that reach up to 166% of their body mass (Reed, 1993) and much of the energy to support these litters comes from the immediate ingestion of food (Kiell and Millar, 1980), all possibly result in Toolik females having elevated FCM levels.

### Reproductive fitness

We found that individual Toolik and Atigun female ground squirrels weaned a similar number of offspring of similar size and condition. Although much of the energy to support reproduction comes from foraging (Kiell and Millar, 1980), we suggest that AGS at the two sites may have different breeding strategies. At both sites, body condition and FCM levels increased from gestation to weaning, yet the body condition of Toolik females only increased to 75% that of Atigun females, and their FCM levels increased to 135% that of Atigun females in 2011. Thus, females at Toolik may tend towards a capital breeding strategy and rely more on internal reserves to support their developing offspring. In contrast, females at Atigun may tend toward an income breeding strategy, where most of their energetic needs for breeding are derived from foraging. Although it may be questioned why Atigun females do not forage more, get bigger and have more offspring, we suggest there is likely a trade-off between the amount of time spent foraging and the risk of predation. It may be a better strategy to minimize risk and live to breed again next year. Survival differences of AGS between the sites remain unknown.

Reproduction can be compromised by glucocorticoid hormone levels (Sheriff *et al.*, 2009), yet we found that female glucocorticoid levels (as indicated by FCM levels) did not seem to affect reproduction at either of our sites. Glucocorticoid levels may modulate female’s ability to mobilize energy differently and cope with the environment. We did find, however, that in 2012 Toolik females had extremely high FCM levels during gestation and their reproductive success was reduced. The number of weaned offspring decreased by >1.5 individuals per female and those that weaned had a lower body condition (relative score of 35 in 2011 vs. −114 in 2012). In comparison, at Atigun in 2012 the number of weaned offspring per female decreased by only 0.6 individuals, and the body condition of offspring increased (relative score of −49 in 2011 vs. −19 in 2012). Thus, we suggest that glucocorticoid hormones may play vital roles in allowing AGS to cope with differences in their phenology-environment relationship, yet if AGS encounter stressors that greatly increase hormone levels it may compromise their reproduction.

### Offspring stress physiology

We found that the patterns of FCM levels in offspring differed between the sites. At weaning, Toolik offspring had similar FCM levels in 2011, but significantly higher levels in 2012 compared to Atigun offspring. At pre-hibernation, Toolik offspring had FCM levels 3 times higher than they had at weaning; whereas Atigun offspring had FCM levels that remained constant and relatively low across these time periods. Given that prior to weaning offspring remain sequestered in their natal burrow and have little experience with the above ground environment, the significantly higher FCM levels in Toolik offspring compared to Atigun offspring at weaning in 2012 may have resulted from the elevated maternal stress during gestation and lactation of adult females at Toolik. Maternal glucocorticoids can influence offspring glucocorticoid levels through a number of mechanisms including pre-natal changes to the offspring’s epigenome and post-natal changes to maternal behavior and offspring care (Meaney *et al.*, 2007; Love *et al.*, 2013; Sheriff *et al.*, 2017).

The elevated levels of FCMs during pre-hibernation in Toolik offspring may reflect their need to fatten and prepare for hibernation in a quickly deteriorating environment. On average female offspring enter hibernation mid-September and males in early October (Sheriff *et al.*, 2011a); however, in early September (when sampling occurred) Toolik was already 25–50% snow covered, reducing offspring’s access to food. In addition to restricted food access in a poor environment, Toolik offspring may then have also needed to expose themselves to riskier situations and engage in dangerous foraging behavior—all of which could increase their FCM levels. Conversely, Atigun offspring would not have needed to engage in such risky foraging behavior since, in early September, Atigun was only 0–5% snow covered. Thus, offspring stress physiology likely result from the indirect and direct influence of their environment; indirectly in spring via their mother’s experience with her environment, and directly in autumn in response to their own experience with the environment.

### Recruitment

We found that pre-hibernation juvenile density was lower and that there was a 10% greater reduction in density from weaning to hibernation at Toolik compared with Atigun. This was particularly evident in 2012, when pre-hibernation density at Toolik was 0.19 juvniles/ha, only 25% of that at weaning, whereas at Atigun the pre-hibernation density was 1.89 juveniles/ha, almost 70% of that at weaning. At weaning in 2012, Toolik offspring were in significantly worse condition and had significantly higher FCM levels, which may have affected their propensity to disperse and their survival (although we do not know the exact fates of juveniles who disappeared off of our trapping grids). Juvenile dispersal in AGS occurs in the first few weeks of July and is sex-specific; studies in the Yukon have shown that females are highly philopatric and disperse approximately 120 m, and males disperse approximately 500 m from the natal area (Byrom and Krebs, 1999). During this time juveniles, regardless of sex, lose significant mass (Buck and Barnes, 1999a); however, little is known about how an individual’s stress physiology may influence dispersal. In other vertebrates, glucocorticoid levels have been shown to alter juvenile dispersal. For example, in willow tits (*Parus montanus*) experimental increases in glucocorticoids increased dispersal propensity (Silverin, 1997), and in the common lizard (*Lacerta vivipara*) experimental increases in both maternal and juvenile glucocorticoids alter juvenile dispersal (Meylan *et al.*, 2004). This has been suggested as an adaptive mechanism increasing offspring movement out of poor habitats (Meylan and Clobert, 2005). Adult density is likely unrelated to the potential greater dispersal of Toolik offspring, given Atigun density is higher than Toolik (see *Reproductive output* in Results).

The low recruitment and large reduction in Toolik offspring density in 2012 may also be driven by decreased offspring survival. In 2012, Toolik offspring were in poor body condition and may have needed to spend more time foraging, increasing their susceptibility to predation (AGS are primary prey for nearly all arctic mammal and avian predators). Additionally, their poor start may have been exacerbated by the poor environment at Toolik, where winter snow begins to accumulate and reduce food availability earlier in the active season. Clearly, the interaction AGS have with their environment likely plays an important role in recruitment of offspring, and as environments change in the spring (altering maternal condition) or in the autumn (altering the ability of offspring to prepare for hibernation) recruitment is likely to be greatly affected.

### Coping with a changing environment

Understanding the potential physiological mechanisms and their fitness consequences that allow animals to cope with rapid environmental changes has been cited as one of the biggest challenges of current biology (Wingfield, 2008, 2013; Németh *et al.*, 2013). Our study is one of the first to integrate marked differences in the relationship between animal phenology and snow cover regimes, with associated differences in foraging opportunities, body condition and stress physiology, reproductive success and recruitment. Our study provides insights into the consequences of changing relationships between animals and their environment. We make the following recommendations for future research:

1. There is accumulating recent evidence that in addition to directional trends and regime shifts, climate-induced environmental changes will include a large increase in extreme weather events (Easterling *et al.*, 2000; Benison and Stephenson, 2004), which can greatly compromise an individual’s survival and reproductive success and, thus, population abundance (Wingfield *et al.*, 2011, 2017; Lane *et al.*, 2012; Krause *et al.* 2016; Williams *et al.*, 2017). We suggest that in the face of climate-induced changes in the relationship between animals and their environment, we must understand that animals that may seemingly be able to adapt strategies to cope with environmental regime shifts may be pushed near the limits of their ability to cope, reducing their perturbation resistance potential (Wingfield *et al.*, 2011).
2. We suggest there is a critical need to also investigate climate-induced changes to autumn conditions. Most studies on environmental change focus on spring conditions, however, we have shown that autumn conditions and the timing of new snow cover influences the spring phenology of both adult male and female AGS (Sheriff *et al.*, 2013, 2015). Here we show that offspring stress physiology and recruitment may also be influenced by autumn conditions and the onset of snow cover. If climate warming, for example, prolongs autumn conditions and delays the onset of snow cover, it could allow offspring more time to prepare for winter, increase recruitment and relax the urgency of early breeding.
3. We provide evidence to support the growing notion that to properly assess the impact of seasonal differences in environmental conditions it is critical to link the animal’s response to multiple fitness metrics (*sensu*Wingfield, 2013). Further, because many estimates of vulnerability are often related to survival, we suggest that we may be underestimating potential climate-induced impacts on populations; i.e. estimates of impacts on survival provide evidence of conditions that are immediately lethal, however, more subtle impacts on reproduction and recruitment may be just as critical and over time contribute greatly to local extirpation.

In conclusion, to better understand how animals may cope with and respond to abiotic stressors in their changing environment, ecologists will need to examine changes at both the individual and population levels; appreciating that predictions based on findings from one level may not translate to another. As we show here, females living in the poorer environment were bigger and in better body condition, had higher stress hormone levels, yet had similar reproductive output as females living in a better environment; at the population level, the poorer environment may have the potential to limit recruitment.

## References

[cox065C1] ACIA (2005) Arctic Climate Assessment. Cambridge University Press, Cambridge.

[cox065C50000] Barnes BM (1989) Freeze avoidance in a mammal: body temperatures below 0 degree C in an Arctic hibernator. Science 244:1593–1594. 2740905 10.1126/science.2740905

[cox065C2] Batzli GO, Sobaski ST (1980) Distribution, abundance, and foraging patterns of ground squirrels near Atkasook, Alaska. Arct Alp Res 12:501–510.

[cox065C3] Benison M, Stephenson DB (2004) Extreme climatic events and their evolution under changing climatic conditions. Glob Planet Change 44:1–9.

[cox065C4] Bonier F, Martin PR, Moore IT, Wingfield JC (2009) Do baseline glucocorticoids predict fitness? Trends Ecol Evol 24:634–642. 19679371 10.1016/j.tree.2009.04.013

[cox065C5] Boonstra R (2004) Coping with changing northern environments: the role of the stress axis in birds and mammals. Integr Comp Biol 44:95–108. 21680490 10.1093/icb/44.2.95

[cox065C6] Boonstra R (2013) Reality as the leading cause of stress: rethinking the impact of chronic stress in nature. Funct Ecol 27:11–23.

[cox065C7] Boonstra R, Hik D, Singleton GR, Tinnikov A (1998) The impact of predator-induced stress on the snowshoe hare cycle. Ecol Monogr 79:371–394.

[cox065C8] Boonstra R, Dantzer B, Delehanty B, Fletcher Q, Sheriff MJ (2014) Equipped for life in the Boreal Forest: the role of the stress axis in mammals. Arctic 67:82–97.

[cox065C9] Both C, Bouwhuis S, Lessells CM, Visser ME (2006) Climate change and population declines in a long-distance migratory bird. Nature 441:81–83. 16672969 10.1038/nature04539

[cox065C10] Both C, Van Turnhout CAM, Bijlsma RG, Siepel H, Van Strien AJ, Foppen RPB (2010) Avian population consequences of climate change are most severe for long-distance migrants in seasonal habitats. Proc R Soc Lond B 277:1259–1266. 10.1098/rspb.2009.1525 PMC2842804 20018784

[cox065C11] Breuner CW, Patterson SH, Hahn TP (2008) In search of relationships between the acute adrenocortical response and fitness. Gen Comp Endocrinol 157:288–295. 18602555 10.1016/j.ygcen.2008.05.017

[cox065C12] Bronson MT (1980) Altitudinal variation in emergence time of golden-mantled ground squirrels (*Spermophilus lateralis*). J Mammal 61:124–126.

[cox065C13] Buck CL, Barnes BM (1999a) Temperatures of hibernacula and changes in body composition of arctic ground squirrels over winter. J Mammal 80:1264–1276.

[cox065C14] Buck CL, Barnes BM (1999b) Annual cycle of body composition and hibernation in free-living arctic ground squirrels. J Mammal 80:430–442.

[cox065C15] Buck CL, Breton A, Kohl F, Tøien Ø, Barnes BM (2008) Overwinter body temperature patterns of free-living arctic ground squirrels (*Spermophilus parryii*) In Lovegrove BG, McKechnie AE (eds) Hypometabolism in Animals: Torpor, Hibernation and Cryobiology. University of KwaZulu-Natal, Pietermaritzburg 317–326.

[cox065C16] Buck CL, O’Reilly KM, Kildaw SD (2007) Interannual variability of Black-legged Kittiwake productivity is reflected in baseline plasma corticosterone. Gen Comp Endocrinol 150:430–436. 17161400 10.1016/j.ygcen.2006.10.011

[cox065C17] Byrom AE, Krebs CJ (1999) Natal dispersal of juvenile artic ground squirrels in the boreal forest. Can J Zool 77:1048–1059.

[cox065C18] Clinchy M, Sheriff MJ, Zanette LY (2013) Predator-induced stress and the ecology of fear. Funct Ecol 27:56–65.

[cox065C20] Dantzer B, Fletcher QE, Boonstra R, Sheriff MJ (2014) Measures of physiological stress: a transparent of opaque window into the status, management and conservation of species. Conserv Physiol 2:cou023 doi:10.1093/conphys/cou023. 27293644 10.1093/conphys/cou023 PMC4732472

[cox065C21] Easterling DR, Meehl GA, Parmesan C, Changon SA, Karl TR, Mearns LO (2000) Climate extremes: observations, modelling and impacts. Science 289:2068–2074. 11000103 10.1126/science.289.5487.2068

[cox065C22] Efford MG, Borchers DL, Byrom AE (2009) Density estimation by spatially explicit capture-recapture: likelihood-based methods In Thomson DL, Cooch EG, Conroy MJ (eds) Modeling Demographic Processes in Marked Populations. Springer, NY 255–269.

[cox065C23] Fauteux D, Gauthier G, Berteaux D, Bosson C, Palme R, Boonstra R (2017) Assessing stress in Arctic lemmings: fecal metabolite levels reflect plasma free corticosterone levels. Physiol Biochem Zool 90:370–382. 28384423 10.1086/691337

[cox065C24] Hobbie EA, Shamhart J, Sheriff MJ, Ouimette AP, Trappe M, Schuur EAG, Hobbie JE, Boonstra R, Barnes BM (2017) Stable isotopes and radiocarbon assess variable importance of plants and fungi in diets of arctic ground squirrels. Arct Antarc Alp Res 49:487–500.

[cox065C25] Inouye DW, Barr B, Armitage KB, Inouye BD (2000) Climate change is affecting altitudinal migrants and hibernating species. Proc Nat Acad Sci 97:1630–1633. 10677510 10.1073/pnas.97.4.1630 PMC26486

[cox065C26] IPCC (2014) Climate Change 2014 In Edenhofer O, et al (eds) Mitigation of Climate Change, Contribution of Working Group III to the Fifth Assessment Report of the Intergovernmental Panel on Climate Change. Cambridge University Press, Cambridge, UK.

[cox065C27] Kiell DJ, Millar JS (1980) Reproduction and nutrient reserves of Arctic ground squirrels. Can J Zool 583:416–421.

[cox065C28] Kitaysky AS, Piatt JF, Hatch SA, Kitaiskaia EV, Benowitz-Fredricks ZM, Shultz MT, Wingfield JC (2010) Food availability and population processes severity of nutritional stress during reproduction predicts survival of long-lived seabirds. Funct Ecol 24:625–637.

[cox065C29] Krause JS, Pérez JH, Chmura HE, Sweet SK, Meddle SL, Hunt KE, Gough L, Boelman N, Wingfield JC (2016) The effects of extreme spring weather on body condition and stress physiology in Lapland longspurs and white-crowned sparrows breeding in the Arctic. Gen Comp Endocrinol 237:10–18. 27449342 10.1016/j.ygcen.2016.07.015 PMC5053339

[cox065C30] Lacey EA (1991) Reproductive and dispersal strategies of male Arctic ground squirrels (*Spermophilus parryii plesius*). PhD Thesis, University of Michigan.

[cox065C31] Lacey EA, Wieczorek JR, Tucker PK (1997) Male mating behaviour and patterns of sperm precedence in Arctic ground squirrels. Anim Behav 53:767–779.

[cox065C32] Lane JE, Kruuk LEB, Charmantier A, Murie JO, Dobson FS (2012) Delayed phenology and reduced fitness associated with climate change in a wild hibernator. Nature 489:554–557. 22878721 10.1038/nature11335

[cox065C33] Love OP, McGowan P, Sheriff MJ (2013) Maternal adversity and ecological stressors in natural populations: the role of stress axis programming in individuals, with implications for populations and communities. Funct Ecol 27:81–92.

[cox065C34] Meaney MJ, Szyf M, Seckl JR (2007) Epigenetic mechanisms of perinatal programming of hypothalamic-pituitary-adrenal function and health. Trends Molec Med 13:269–277. 17544850 10.1016/j.molmed.2007.05.003

[cox065C35] Meylan S, Clobert J (2005) Is corticosterone-mediated phenotype development adaptive? Maternal Corticosterone treatment enhances survival in male lizards. Horm Behav 48:44–52. 15919384 10.1016/j.yhbeh.2004.11.022

[cox065C36] Meylan S, de Fraipont M, Clobert J (2004) Maternal size and stress and offspring philopatry: an experimental study in the common lizard (*Lacerta vivipara*). Ecoscience 11:123–129.

[cox065C37] Meylan S, Miles DB, Clobert JR (2012) Hormonally mediated maternal effects, individual strategy and global change. Phil Trans R Soc B 367:1647–1664. 22566673 10.1098/rstb.2012.0020 PMC3350661

[cox065C38] Montanholi YR, Palme R, Haas LS, Swanson KC, Van der Voort G, Miller SP (2013) On the relationship between glucocorticoids and feed efficiency in beef cattle. Livestock Sci 155:130–136.

[cox065C39] Möstl E, Maggs JL, Schrötter G, Besenfelder U, Palme R (2002) Measurement of cortisol metabolites in faeces of ruminants. Vet Res Commun 26:127–139. 11922482 10.1023/a:1014095618125

[cox065C40] Murie JO, Harris MA (1982) Annual variation of spring emergence and breeding in Columbian ground squirrels (*Spermophilus columbianus*). J Mammal 63:431–439.

[cox065C41] Naughton D (2012) The Natural History of Canadian Mammals. University of Toronto Press, Toronto, ON.

[cox065C42] Németh Z, Bonier F, MacDougall-Shackleton SA (2013) Coping with uncertainty: integrating physiology, behavior, and evolutionary ecology in a changing world. Integr Comp Biol 53:960–964. 23933811 10.1093/icb/ict089

[cox065C43] Ozgul A, Childs DZ, Oli MK, Armitage KB, Blumstein DT, Olson LE, Tuljapurkar S, Coulson T (2010) Coupled dynamics of body mass and population growth in response to environmental change. Nature 466:482–485. 20651690 10.1038/nature09210 PMC5677226

[cox065C44] Palme R, Rettenbacher S, Touma C, El-Bahr SM, Möstl E (2005) Stress hormones in mammals and birds: comparative aspects regarding metabolism, excretion and noninvasive measurement in fecal samples. Trends in Comparative Endocrinology and Neurobiology. Annals New York Acad Sci 1040:162–171. 10.1196/annals.1327.021 15891021

[cox065C45] Parmesan C, Yohe G (2003) A globally coherent fingerprint of climate change impacts across natural systems. Nature 421:37–42. 12511946 10.1038/nature01286

[cox065C46] Pörtner HA, Farrell AP (2008) Physiology and climate change. Science 31:690–692. 10.1126/science.1163156 18974339

[cox065C40000] Post E, Forchhammer MC (2008) Climate change reduces reproductive success of an Arctic herbivore through trophic mismatch. Phil Trans R Soc Lond B 363:2369–2375. 18006410 10.1098/rstb.2007.2207 PMC2606787

[cox065C47] Reed MC (1993) Growth in arctic ground squirrels and the influence of a cache on hibernation and reproductive maturation. Master’s Thesis, Department of Biology and Wildlife, University of Alaska Fairbanks, Fairbanks, Alaska, USA.

[cox065C48] Romero LM (2004) Physiological stress in ecology: lessons from biomedical research. Trends Ecol Evol 19:249–255. 16701264 10.1016/j.tree.2004.03.008

[cox065C49] Romero LM, Reed JM, Wingfield JC (2000) Effect of weather on corticosterone responses in wild free-living passerine birds. Gen Comp Endocrinol 118:113–122. 10753573 10.1006/gcen.1999.7446

[cox065C50] Satherthwaite WH, Kitayski AS, Mangel M (2012) Linking climate variability, productivity and stress to demography in a long-lived seabird. Mar Ecol Pr Ser 454:221–235.

[cox065C51] Saino N, Ambrosini R, Rubolini D, von Hardenberg J, Provenzale A, Hüppop K, Hüppop O, Lehikoinen A, Lehikoinen E, Rainio K, Romano M, et al (2011) Climate warming, ecological mismatch at arrival and population decline in migratory birds. Proc R Soc Lond B 278:835–842. 10.1098/rspb.2010.1778 PMC3049050 20861045

[cox065C52] Sapolsky RM, Romero LM, Munck AU (2000) How do glucocorticoids influence stress responses? Integrating permissive, suppressive, stimulatory, and preparative actions. Endocr Rev 21:55–89. 10696570 10.1210/edrv.21.1.0389

[cox065C53] Selye HA (1936) A syndrome produced by diverse nocuous agents. Nature 138:32–33. 10.1176/jnp.10.2.230a 9722327

[cox065C54] Schulte-Hostedde AI, Zinner B, Millar JS, Hickling GJ (2005) Restitution of mass-size residuals: validating body condition indices. Ecology 86:155–163.

[cox065C55] Sheriff MJ, Krebs CJ, Boonstra R (2009) The sensitive hare: sublethal effects of predator stress on reproduction in snowshoe hares. J Anim Ecol 78:1249–1258. 19426257 10.1111/j.1365-2656.2009.01552.x

[cox065C56] Sheriff MJ, Krebs CJ, Boonstra R (2010) Assessing stress in animal populations: do fecal and plasma glucocorticoids tell the same story? Gen Comp Endocrinol 166:614–619. 20051245 10.1016/j.ygcen.2009.12.017

[cox065C57] Sheriff MJ, Kenagy GJ, Richter M, Lee T, Tøien Ø, Kohl F, Buck CL, Barnes BM (2011a) Phenological variation in annual timing of hibernation and breeding in nearby population of Arctic ground squirrels. Proc R Soc B 278:2369–2375. 10.1098/rspb.2010.2482 PMC3119018 21177687

[cox065C58] Sheriff MJ, Dantzer B, Delehanty B, Palme R, Boonstra R (2011b) Measuring stress in wildlife: techniques for quantifying glucocorticoids. Oecologia 166:869–887. 21344254 10.1007/s00442-011-1943-y

[cox065C59] Sheriff MJ, Wheeler H, Donker SA, Krebs CJ, Palme R, Hik DS, Boonstra R (2012) Mountain‐top and valley‐bottom experiences: the stress axis as an integrator of environmental variability in arctic ground squirrel populations. J Zool 287:65–75.

[cox065C60] Sheriff MJ, Richter MM, Buck CL, Barnes BM (2013) Changing seasonality and phenological responses of free-living male artic ground squirrels: the importance of sex. Phil Trans R Soc B 368:20120480. 23836786 10.1098/rstb.2012.0480 PMC3720053

[cox065C61] Sheriff MJ, Buck CL, Barnes BM (2015) Autumn conditions as a driver of spring phenology in a free-living arctic mammal. Clim Change Res 2:4 doi:10.1186/s40665-015-0012-x.

[cox065C62] Sheriff MJ, Bell A, Boonstra R, Dantzer B, Lavergne S, McGhee KE, MacLeod KJ, Winandy L, Zimmer C, Love OP (2017) Integrating ecological and evolutionary context in the study of maternal stress. Integr Comp Biol 57:437–449. 28957523 10.1093/icb/icx105 PMC5886325

[cox065C30000] Silverin B (1997) The stress response and autumn dispersal behaviour in willow tits. Anim Behav 53:451–459.

[cox065C63] Visser ME, van Noordwijk A, Tinbergen JM, Lessells CM (1998) Warmer spring lead to mistimed reproduction in great tits (*Parus major*). Proc R Soc B 265:1867–1870.

[cox065C64] Werner JR, Krebs CJ, Donker SA, Boonstra R, Sheriff MJ (2015) Arctic ground squirrel population collapse in the boreal forests of the southern Yukon. Wildl Res 42:176–184.

[cox065C65] Wikelski M, Cooke SJ (2006) Conservation physiology. Trends Ecol Evol 21:38–46. 16701468 10.1016/j.tree.2005.10.018

[cox065C66] Williams CT, Barnes BM, Kenagy GJ, Buck CL (2014) Phenology of hibernation and reproduction in ground squirrels: integration of environmental cues with endogenous programming. J Zool 292:112–124.

[cox065C67] Williams CT, Buck CL, Sheriff MJ, Richter MM, Krause JS, Barnes BM (2017) Sex-dependent phenological plasticity in an arctic hibernator. Am Nat 190, doi.org/10.1086/694320. 10.1086/694320 29166160

[cox065C68] Wingfield JC (2008) Comparative endocrinology, environment and global change. Gen Comp Endocrinol 157:207–216. 18558405 10.1016/j.ygcen.2008.04.017

[cox065C69] Wingfield JC (2013) The comparative biology of environmental stress: behavioural endocrinology and variation in ability to cope with novel, changing environments. Anim Behav 85:1127–1133.

[cox065C70] Wingfield JC, Maney DL, Breuner CW, Jacobs JD, Lynn S, Ramenofsky M, Richardson RD (1998) Ecological bases of hormone-behavior interactions: the ‘emergency life history stage’. Am Zool 38:191–206.

[cox065C71] Wingfield JC, Kelley JP, Angelier F (2011) What are the extreme environmental conditions and how do organisms cope with them? Current Zool 57:363–374.

[cox065C72] Wingfield JC, Pérez JH, Krause JS, Word KR, González-Gomez, Lisovski S, Chmura HE (2017) How birds cope physiologically and behaviorally with extreme climatic events. Phil Trans R Soc B 372:20160140. 28483870 10.1098/rstb.2016.0140 PMC5434091

